# Echocardiographic estimation of left ventricular filling pressures in heart transplant recipients

**DOI:** 10.1186/s43044-024-00443-z

**Published:** 2024-01-30

**Authors:** Zohreh Rahbar, Anahita Tavoosi, Alireza Bakhshandeh, Maryam Mehrpooya, Akram Sardari, Farnoosh Larti, Roya Sattarzadeh Badkoubeh

**Affiliations:** 1 Department of Cardiology, Imam Khomeini Hospital Complex, Tehran University of Medical Sciences, End of Keshavarz Boulevard, Tehran, 1419733141 Iran; 2 Department of Cardiothoracic Surgery, Imam Khomeini Hospital Complex, Tehran University of Medical Sciences, Tehran, Iran

**Keywords:** Echocardiography, Heart transplantation, LVEDP, Diastolic dysfunction, *E*/*e*′, GLS

## Abstract

**Background:**

Echocardiographic estimation of left ventricular filling pressure in heart transplant (HTx) recipients is challenging. The ability of echocardiography to detect elevated left ventricular end-diastolic pressure (LVEDP) in HTx patients was assessed in this study.

**Results:**

This descriptive cross-sectional study included 39 HTx recipients who were candidates for endomyocardial biopsy as a part of their routine post-transplantation surveillance. Doppler transthoracic echocardiography was done before the procedure, and left heart catheterization was done during the endomyocardial biopsy. Thirty-nine patients (15 female, 24 male), with a mean age of 39.6 years (range 13–70), were enrolled. A strong relation was observed between lateral *E*/*e*′ and LVEDP (*R* = 0.64, *P* value < 0.001) and average *E*/*e*′ and LVEDP (*R* = 0.6, *P* value < 0.001). The best cutoff value for LVEDP prediction was the average *E*/*e*′ ≥ 6.8 with a sensitivity of 96.15% and specificity of 68.5% for the prediction of LVEDP more than or equal to 20 mmHg. Two predictive models comprising age, gender, and lateral *E*/*e*′ or average *E*/*e*′ were also proposed. A significant relationship was also found between LVEDP and left ventricular global longitudinal strain (*R* = − 0.31, *P* value < 0.01).

**Conclusions:**

Lateral *E*/*e*′ was the best predictor of LVEDP. The cutoff of average *E*/*e*′ had the best validity for the estimation of LVEDP. Despite the strong observed association, echocardiographic parameters cannot be considered a surrogate for invasive LVEDP measurements when seeking information about left ventricle filling pressure on heart transplant recipients.

## Background

Diastolic dysfunction is a common finding early after heart transplantation (HTx). It may be caused by preoperative ischemic [1], low-grade inflammation, fibrosis [2], and rarely cardiac allograft vasculopathy [3]. Several weeks after transplantation, the elevated filling pressure will decrease. Recurrence of diastolic dysfunction later is due to rejection episodes, hypertension, and myocardial ischemia from cardiac allograft vasculopathy. In the first year after transplantation and patients with acute rejection, diastolic dysfunction is considered a risk factor for increased mortality [4]. The results of studies that assessed left ventricular filling pressure by echocardiography in HTx patients are conflicting [5, 6]. Increased left ventricle filling pressure on catheterization is often defined as LV end-diastolic filling pressure > 16 mmHg or pulmonary capillary wedge pressure (PCWP) > 12 mmHg [7–9]. In echocardiography, measurement of mitral inflow and pulmonary veins velocities has yet to be demonstrated to predict pulmonary capillary wedge pressure (PCWP) in transplant recipients field [6, 10–13], and tissue Doppler measurements are shown useful by some investigators but not by the two recent recommendations for evaluating diastolic dysfunction need to provide clear guidance regarding determining left ventricular end-diastolic pressure (LVEDP) in HTx recipients [4, 10, 14–16].

Atrial function is affected considerably in HTx patients. Echocardiographic indices estimating LV filling pressure highly depend on the left atrial function [17, 18]. Because PCWP is affected by atrial pressure and function and considering the challenges of measuring PCWP in previous studies, we focused on LVFEDP in this study. The ability of echocardiography to detect elevated left ventricular end-diastolic pressure (LVEDP) in HTx patients was assessed.

## Methods

### Study population

This descriptive cross-sectional single-center study comprised 39 consecutive HTx recipients investigated with Doppler echocardiography and left heart catheterization (LHC) at our institute from May 2016 to February 2018. Consecutive patients who were candidates for myocardial biopsy (as part of their routine post-transplantation surveillance) were included in the study. Informed consent for myocardial biopsy was obtained, and the in-charge attending physician explained the procedure to the patient. The detailed study process was discussed thoroughly with each patient, and informed consent was obtained. The ethics committee of our institute approved the study.

The inclusion criteria comprised HTx recipients with the bi-caval method who were in sinus rhythm. The study protocol consisted of performing detailed echocardiography at the first step and then left heart catheterization, with a time gap of no more than 12 h between echocardiography and catheterization. The exclusion criteria were refusal to participate in the study, presence of arrhythmia including atrial fibrillation, and any echocardiographic findings that interfere with assessing LV diastolic dysfunction (e.g., more than moderate mitral regurgitation). STROBE cross-sectional reporting guidelines were used to report the study results [19].

### Echocardiography

Echocardiographic evaluation after cardiac transplantation was performed with Philips (Affiniti 50C) ultrasound machine using a phased array transducer and with 3.0.3 Qlab11 and Automated Cardiac Motion Quantification (aCMQ) software. All echocardiographic exams were performed and analyzed by one operator blind to invasive LHC results. Two-dimensional and conventional Doppler parameters were performed according to current recommendations, and valvular regurgitations were graded as mild, moderate, or severe [14, 20, 21]. The left ventricular end-diastolic and systolic volumes were measured, and ejection fractions were calculated using bi-plane Simpson’s method.

Early mitral inflow velocity (*E*) was obtained by pulsed-wave Doppler at the tip of the mitral leaflets. Mitral annular early diastolic velocity (*e*′) of septal and lateral walls was obtained from color-coded tissue Doppler imaging (TDI) images. A 10-mm sample volume was positioned at the septal and lateral insertion of the mitral valve, respectively. *E* wave velocity was divided by *e*′ measured in the septum (*e*′ septal) and lateral wall (*e*′ lateral) in the four-chamber view [22] to measure septal *E*/*e*′ and lateral *E*/*e*′, respectively. The average septal *e*′ and lateral *e*′ were calculated, and average *E*/*e*′ was defined as *E* divided by average *e*′.

**Fig. 1:**
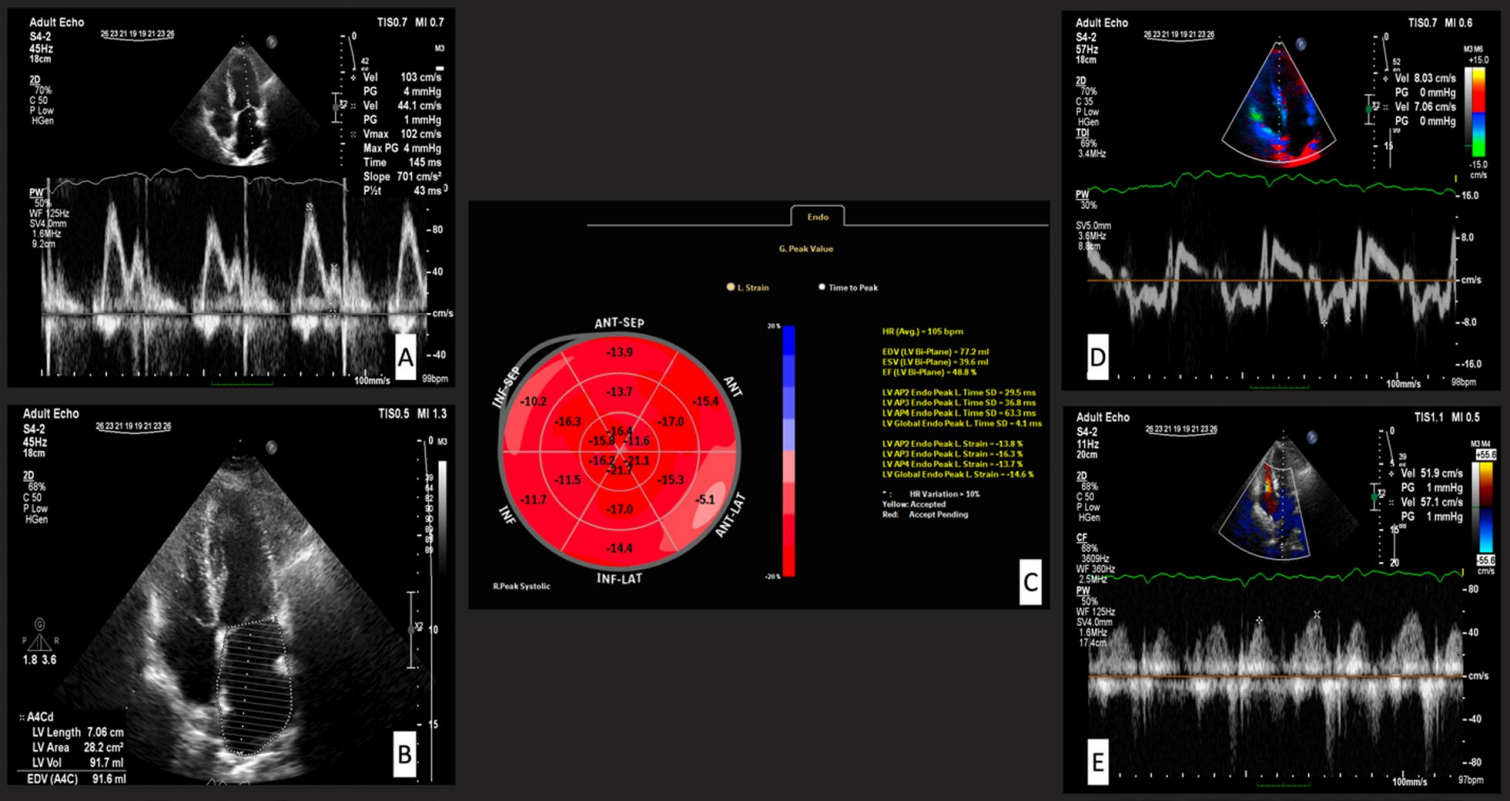
**A** Early mitral inflow velocity (*E*), Late mitral inflow velocity (*A*), and deceleration time (DT) by pulsed-wave Doppler at the tip of the mitral leaflets; **B** Left atrial area and volume in 4-chamber view; **C** Global longitudinal strain (GLS) of LV; **D** Mitral annular early diastolic velocity (*e*′) and late diastolic velocity (*a*′) of the septal wall from color-coded tissue Doppler imaging (TDI) image; **E** Pulmonary vein peak velocity in systole and diastole

Global longitudinal strain (GLS) was assessed online with an EPIQ 7 Ultrasound machine using aCMQ software. Peak longitudinal systolic strain in apical 2-chamber, apical 4-chamber, and apical 3-chamber views was measured online using three stored image loops. The operator visually assessed the software’s accuracy in tracking the ventricular motion and made necessary changes manually [23]. In Fig. 1, some measured echocardiographic indices were shown. Depending on the working schedule of attending physicians in our echocardiography lab, the EPIQ 7 Ultrasound machine was only available for research projects on certain days of the week, so we could not measure GLS in every patient before LHC.

### Left heart catheterization

Before LHC, all patients received 500 ml of isotonic fluid intravenously. The LHC was performed in a fasting state using only local anesthesia at the needle puncture site.

A 7F fluid-filled catheter was placed in the LV by a femoral percutaneous approach. The fluid-filled pressure was calibrated with the external pressure transducer at the mid-axillary level. All the recordings were done before the injection of the contrast agent. The LVEDP was measured at the nadir of the atrial contraction wave before a rapid rise in LV systolic pressure. In cases without a clear atrial contraction wave, LVEDP has obtained 50 ms after the beginning of the QRS complex. Hemodynamic data were collected by an investigator unaware of the echocardiographic measurements and represented the average of five cycles. The relation between echocardiographic data and invasive LVEDP was evaluated in our study.

### Statistical analysis

Mean (SD) and median (interquartile range; IQR) were calculated for descriptive data. The normality of data was tested by one sample Kolmogorov–Smirnov test. An independent t test was used to compare LVEDP levels in males and females. Correlations were identified by Spearman’s and Pearson’s correlation analyses. Absolute correlation values of 0–0.19 were interpreted as very weak or no correlation, 0.20–0.39 as weak, 0.40–0.59 as moderate, 0.60–0.79 as strong, and 0.80–1.00 as robust. Next, a series of multivariate linear regression models for predicting LVEDP by echocardiographic parameters were estimated; robust estimators accounted for deviations from normality. Multiple imputations were done for handling missing data using Stata 14. All statistical analysis was performed by Stata software, version 14. *P* values < 0.05 were considered to represent statistically significant relationships.

## Results

### Baseline data

A total of 39 patients participated in this study. Thirteen patients (33%) were female. The mean age was 39.6 ± 12.8 years (range 13–70). The mean years after transplantation were 3.3 ± 2.2 years (0.5–8 years). Table 1 shows echocardiographic parameters and correlation to LVEDP in these patients. Twenty (51%) patients had LVEF less than 50%. The average LVEDP was 18.1 ± 5.9 mmHg. The mean (SD) of LVEDP in females was higher than in males [20.0 (7.0) and 17.4 (5.7); *P* < 0.001]. In thirty-one patients (79%), LVEDP was more than 15 mmHg.

**Table 1:** Echocardiographic parameters of heart transplant recipients investigated for prediction of LVEDP

	Number	Min	Max	Mean (SD)	Median (IQR)	Correlation Coefficient
Age (yrs)	39	13	70	39.6 (12.9)	39 (30–48)	0.23**
EF (%)	39	30	69.1	47.2 (10.4)	49.2 (37.7–54.4)	− 0.16^**^
GLS (%)	31	7.0	18.4	14.0 (3.1)	14.9 (11.4–16.7)	− 0.22**
LVEDP (mmHg)	39	8	33	18.2 (5.9)	18 (15–20)	–
*E* velocity (m/s)	39	0.39	1.1	0.7 (0.2)	0.8 (0.5–0.8)	0.26***
*A* velocity (m/s)	34	0.34	0.98	0.55 (0.18)	0.51 (0.37–0.71)	0.17
*E*/*A*	34	0.02	2.4	1.27 (0.48)	1.12 (1.01–1.48)	− 0.06
DT (msec)	37	56.0	284.0	170.1 (47.9)	169 (140.1–201.0)	0.10
S velocity (cm/s)	32	0.2	0.6	0.4 (0.1)	0.4 (0.3–0.4)	− 0.40***
D velocity (cm/s)	32	0.2	0.9	0.5 (0.2)	0.5 (0.4–0.6)	− 0.27***
*A* reversal (cm/s)	5	0.2	1.94	0.57 (0.77)	0.22 (0.21–1.1)	0.32
S/(S + D)	32	0.3	0.7	0.4 (0.1)	0.4 (0.4–0.5)	− 0.02
Septal *e*′ (cm/s)	39	4.5	13.8	8.8 (3.43)	8.3(6.0)	− 0.09
Septal *a*′(cm/s)	38	3.0	12.4	6.9(2.2)	6.5(5.2–7.4)	− 0.36***
Septal *E*/*e*′	39	4.0	20.0	10.0(4.0)	9.1(7.0–14.0)	0.22**
Lateral *e*′(cm/s)	38	6.3	19.0	11.4 (3.25)	11.0 (9.6–13.0)	− 0.27**
Lateral *a*′(cm/s)	35	3.0	11.6	6.9 (2.39)	6.6 (5.0–8.8)	0.27**
Lateral *E*/*e*′	33	3.0	14.0	6.0 (3.0)	5.0 (4.0–8.0)	0.64***
Average *E*/*e*′	37	4.0	16.0	8.0 (3.0)	6.0(5.0–9.0)	0.60***
LA area (cm^2^)	39	10.8	29.9	18.7 (7.8)	16.9 (14.6)	0.17*
LA volume (cm^3^)	37	24.6	122.0	63.2 (21.9)	62.0 (51.2–73.5)	0.52***

LVEF = left ventricle ejection fraction, GLS = global longitudinal strain, LA = left atrium, Ar = pulmonary venous flow reversal, *E*/*A* = The ratio of peak velocity flow in early diastole [the *E* wave(m/s)] to peak velocity flow in late diastole caused by atrial contraction [the *A* wave(m/s)], *E*/*e*′ = The ratio of early mitral inflow velocity to mitral annular early diastolic velocity, *e*′ (cm/s) = mitral annular early diastolic velocity, *a*′ (cm/s) = mitral annular late diastolic velocity, DT(msec) = Deceleration time, S(cm/s) = pulmonary vein peak systolic velocity, D(cm/s) = pulmonary vein peak diastolic velocity

^*^P value < 0.05, **P value < 0.01, *** *P* value < 0.001

### Correlation of LVEDP to echocardiography parameters

**Fig. 2:**
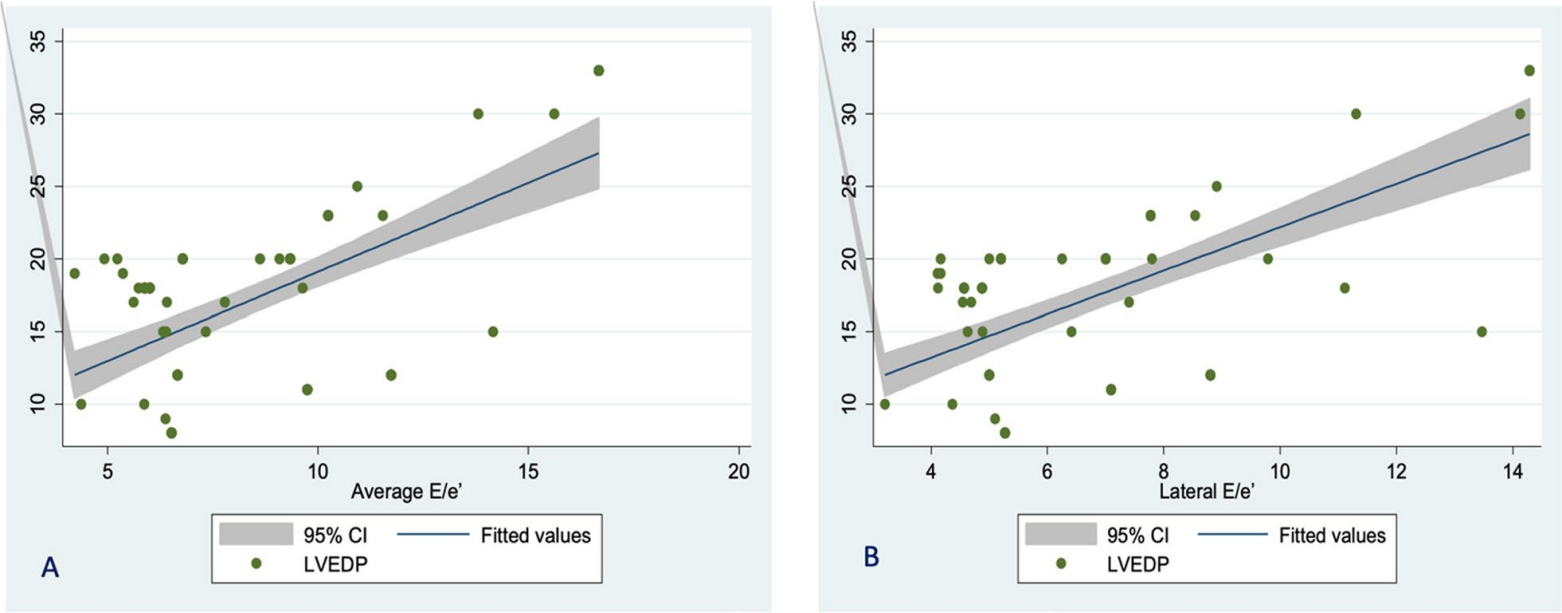
Scatter plot showing the linear relation between LVEDP and average *E*/*e*′ (**A**) and lateral *E*/*e*′ (**B**)

There was a significant (although weak) positive linear correlation between LVEDP and *E* velocity, *A* velocity, and LA area, respectively. A moderate correlation was found between LVEDP and septal *E*/*e*′ and LA volume. Lateral *E*/*e*′ and average *E*/*e*′ had a strong correlation with LVEDP. On the other hand, there was a negative moderate significant linear correlation between LVEF, GLS, deceleration time (DT), septal *e*′, septal *a*′, and LVEDP. Figure 2 shows a linear relationship between lateral *E*/*e*′ and average *E*/*e*′ with LVEDP.

Two models were produced by multiple linear regression analysis to predict LVEDP. Reference model 1 comprised age, gender, and average *E*/*e*′, while reference model 2 included age, gender, and lateral *E*/*e*′. The highest R^2^ value was obtained from model 2 using lateral *E*/*e*′ with R^2^ = 0.64, followed by model 1 using average *E*/*e*′ with R^2^ = 0.60. Detailed data regarding which variables have been used to construct each predictive model are provided in Appendix 1. Regression equations were calculated with these two formulae (there is no need to convert the *E* and *e*′ units, and obtained echocardiographic data should be used in both formulae directly):

$${\text{ LVEDP}} = - 0.99 + \left[ {0.23 \times {\text{Age}}\left( {{\text{year}}} \right)\left] - \right[1.8 \times {\text{Gender}}\left] + \right[185.83 \times {\text{lateral E}}/{\text{e}}^{\prime } } \right]$$

$${\text{ LVEDP}} = - 2 + \left[ {0.23 \times {\text{Age}}\left( {{\text{year}}} \right)\left] - \right[1.9 \times {\text{Gender}}\left] + \right[157.17 \times {\text{average E}}/{\text{e}}^{\prime } } \right]$$

Gender: Female = 1, Male = 2, *E*: early trans-mitral flow velocity (m/s), Lateral *e*′: lateral annular velocity (cm/s), Average *e*′: average of annular velocities (cm/s).

**Fig. 3:**
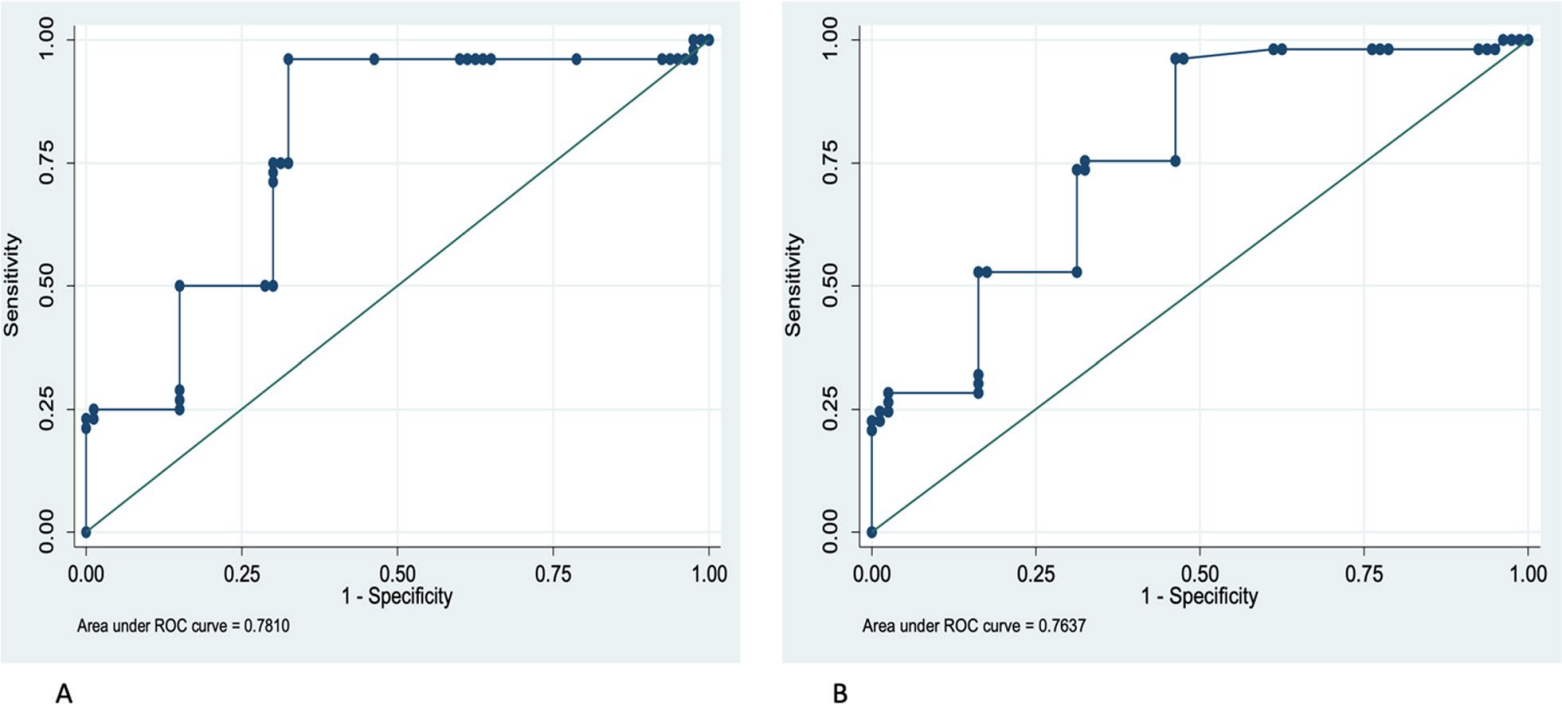
**A** ROC curve for Average *E*/*e*′. Receiver operating characteristic (ROC) curves to detect the diagnostic performance of *E*/*e*′ for predicting LVEDP more than 20 mmHg. Average *E*/*e*′ = the ratio between early mitral inflow velocity and average septal and lateral mitral annular early diastolic velocity. LVEDP, Left ventricular end-diastolic pressure; AUC, area under the curve; CI, confidence interval. AUC = 0.78 (% 95 CI 0.70–0.86), Cut point = 6.8; Sensitivity = 96.15%; Specificity = 67.5%; correctly classification = 78.7%. **B** ROC curve for lateral *E*/*e*′. Receiver operating characteristic (ROC) curves to detect lateral *E*/*e*′s diagnostic performance for predicting LVEDP more than 20 mmHg. Lateral *E*/*e*′ = the ratio between early mitral inflow velocity and lateral mitral annular early diastolic velocity. AUC = 0.76 (95% CI 0.68–0.84), Cut point = 6.2; Sensitivity = 75.47%; Specificity = 67.5%; Correctly classification = 70.68%)

To determine the best cutoff value of lateral *E*/*e*′ and average *E*/*e*′, we assessed two different LVEDP values. First, we considered a cutoff of 16 mmHg for LVEDP with no predictive power. Subsequently, LVEDP above or equal to 20 mm Hg was chosen. Figure 3 shows ROC curves for lateral *E*/*e*′ and average *E*/*e*′. Average *E*/*e*′ more than or equal to 6.8 identified patients with LVEDP $\ge$ 20 mmHg with a sensitivity of 96.15% and specificity of 68.5%. The area under the curve was 0.78 (%95 CI 0.79–0.86), which could correctly classify patients with LVEDP $\ge$ 20 mmHg in 78.79% of cases (Fig. 3A). Figure 2B shows that lateral *E*/*e*′ = 6.2 can predict LVEDP more than or equal to 20 mmHg with AUC = 0.76 (95% CI 0.68–0.84), a sensitivity of 75.47% and specificity of 67.5%, that could correctly classify patients with LVEDP $\ge$ 20 mmHg in 70.68% of cases.

## Discussion

In the present study, we evaluated HTx recipients and found that echocardiography can be used to assess left ventricular filling pressure. Until now, a few studies have been done in HTx recipients with a small number of patients that explored left-sided or right-sided filling pressures [22].

Based on previous studies, the European Society of Echocardiography and the American Society of Echocardiography recommend using average *E*/*e*′ in algorithms for diagnosing diastolic dysfunction. Values of 14 or higher are considered an elevated LV filling pressure [10]. It should be highlighted that the average *E*/*e*′ cutoff value of 14 is based on pulsed-wave-derived tissue Doppler velocities and has not been validated in HTx recipients.

Our findings demonstrated that lateral *E*/*e*′ and average *E*/*e*′ were the only echocardiographic parameters with the highest correlation with LV filling pressure. Other echocardiographic parameters with moderate correlation with LVEDP include septal *E*/*e*′, septal *e*′, lateral *a*′, deceleration time, and LA volume. We also find a moderate negative correlation between the systolic parameters (LVEF, GLS) and LVEDP. Our study produced the best models for LVEDP prediction in HTx patients by combining age, gender, and lateral *E*/*e*′ (R^2^ = 0.64) or average *E*/*e*′ (R^2^ = 0.60)$.$ Due to baseline tachycardia, measurement of *A* wave and annular *a*′ is not always feasible in HTx recipients. So, proposed models without dependency on the late diastolic echocardiographic parameters seem practical.

Interestingly, adding LA volume to the prediction model did not improve the model. Average *E*/*e*′ more than 6.8 could correctly classify patients with LVEDP as more than or equal to 20 mmHg in 78.7% of our HTx recipients with a sensitivity of 96.15% and specificity of 68.5%. This threshold is considerably lower than the recommended threshold in non-HTX patients; one explanation for this observation could be the high prevalence of elevated LVEDP in our patients. The other important reason may be the different invasive methods of measuring LV filling pressure compared to previous research. PCWP measurement is moderately correlated with LVEDP in heart transplantation, and measuring PCWP may lead to misclassification of pulmonary hypertension in OHT patients [24]. Direct measurement of LVEDP may be the advantage of the presenting study. Despite the recommendation for using septal *E*/*e*′ in non-HTx patients, the correlation between septal *E*/*e*′ and LVEDP was not strong in HTx patients in our study. Although LVEF and GLS are echocardiographic parameters relating to systolic function, they had a moderate negative correlation with LVEDP in this research. From a pathophysiological perspective, loss of myocardial function and increased myocardial fibrosis may result in impaired LV systolic function, decreased LV relaxation, and increased LV stiffness, yet in echocardiographic assessment of LV diastolic function, measures of LV systolic function routinely have no apparent impact.

*E*/*e*′ is a widely accepted noninvasive parameter for anticipating LV filling pressure [25]. *E* velocity depends on LV relaxation and filling pressure, while *e*′ is related to LV relaxation. Theoretically, the *E*/*e*′ index primarily shows LV filling pressure. However, other factors, including ventricular and atrial compliance, LV systolic function, and mitral valve features, may also affect *E* velocity. Similarly, *e*′ relates to LV relaxation, filling pressure, and systolic LV shortening [26]. In the non-HTx study population, Ommen et al. reported a higher correlation coefficient between septal *E*/*e*′ and LVEDP (0.64) relative to average *E*/*e*′ and LVEDP (0.62) [27]. A wide range of correlation coefficients has been reported for the association between *E*/*e*′ and invasively obtained PCWP (0.18–0.8) with most groups reporting intermediate correlations [27, 28]. Despite multiple studies that proposed *E*/*e*′ as a marker of LVEDP estimation, there is still a significant gap in using this echocardiographic parameter as a substitute for LV filling pressure, especially in patients with preserved LVEF, hypertrophic cardiomyopathy, and after cardiac surgery [14, 29].

Regarding different study populations with diverse LV systolic function and underlying pathology, finding a single *E*/*e*′ cutoff with considerable efficacy for all the patients is challenging. The study population mentioned above mainly consisted of non-HTx heart failure patients. The change in septal function and geometry and the altered atrial function and geometry may affect LA pressure post-heart transplantation with a resultant shift in the *E*/*e*′ threshold.

In a study on HTx recipients, atrial reservoir function in LA speckle echocardiography is markedly reduced and related to elevating PCWP [5]. Still, as we did, a study has yet to investigate the relationship between GLS and left ventricular end-diastolic pressure. Because LV systolic and diastolic functions are tightly coupled [7], future research to clarify this association seems mandatory.

This study used a specific protocol and high-standard equipment to minimize errors. However, measuring some echocardiographic indices, such as pulmonary vein Ar was impossible in many patients. One major limitation is that echocardiography and left-sided heart catheterization were not performed simultaneously. However, the time gap between these two procedures was up to 12 h, and all the patients were clinically stable on the evaluation day with no expected considerable changes in hemodynamics. GLS measurement was only feasible sometimes for some patients during working hours in our echocardiography lab. We used the multiple imputations method for handling missing data, and this procedure has some uncertainty due to imputations (limitation of covariates adopted in the imputation model). The number of patients with elevated LVEDP was high in our study group. This could affect the coefficient for the correlation between *E*/*e*′ and LVEDP. Future research with a wide range of LVEDP in HTx recipients can elucidate the reproducibility of these data.

## Conclusions

We found that echocardiography can predict LV filling pressures in HTx recipients. The most reliable index for the estimation of LVEDP was lateral *E*/*e*′, which had a strong association with LVEDP, followed by average *E*/*e*′. Despite these appealing associations, till now, echocardiographic data cannot be used by sure to estimate left ventricle filling pressure in HTx recipients. Extrapolation of the presenting study results may not be possible for general HTx patients, and many factors, including LVEF and time after transplantation, may affect this prediction.

## Data Availability

The study’s data are available for further analysis upon the reasonable request of the corresponding author.

## Appendix 1: Various models produced by multiple linear regression analysis for the prediction of LVEDP

**Table:**

	Model 1	Model 2	Model 3	Model 4	Model 5	Model 6	Model 7
Age	0.23***	0.23***	0.15***	0.14***	0.11***	0.15**	0.21***
Gender	− 1.9**	− 1.8*	0.11	0.68	1.09	0.73	− 0.43
Average*E*/*e*′	157.17***		118.33***	111.46***		120.49***	143.38***
Lateral *E*/*e*′		185.83***					
Septal *E*/*e*′					46.93**		
LA volume				0.06*	0.08**	0.06*	0.07**
Septal *a*′			− 0.76*	− 0.68*	− 0.74*	− 0.67*	− 0.76**
GLS						0.15	
LVEF							0.21***
Intercept	− 1.96*	− 0.99*	6.65**	1.96	5.51	− 1.34	− 10.44*
Model F	62.89	77.79	13.14	15.33	11.44	11.76	17.6
*R*^2^, %	60	64.4	33	45.18	38.09	45.35	53.44

Values are coefficients. Robust estimators are used. Boldface indicates statistical significance (**P* < 0.05; ***P* < 0.01; ****P* < 0.001)

LVEDP, Left ventricular end-diastolic pressure, *E*/*e*′, the ratio between early mitral inflow velocity and mitral annular early diastolic velocity, LA, left atrium, *e*′, mitral annular early diastolic velocity, *a*′, mitral annular late diastolic velocity, GLS, global longitudinal strain, LVEF, left ventricle ejection fraction

## Abbreviations

HTx: Heart transplant
LVEDP: Left ventricular end-diastolic pressure
LA: Left atrium
PCWP: Pulmonary capillary wedge pressure
LHC: Left heart catheterization
aCMQ software: Automated Cardiac Motion Quantification software
*E*: Early mitral inflow velocity
*A*: Late mitral inflow velocity
*e*′: Mitral annular early diastolic velocity
TDI: Tissue Doppler imaging
GLS: Global longitudinal strain
IQR: Interquartile range
DT: Deceleration time
Ar: Pulmonary venous flow reversal
*E*/*A*: Ratio of peak velocity flow in early diastole to peak velocity flow in late diastole
*E*/*e*′: Ratio of early mitral inflow velocity to mitral annular early diastolic velocity
